# Environmental stability of untreated neutral metalliferous mine drainage sludge after 100 years of weathering

**DOI:** 10.1007/s10653-025-02865-3

**Published:** 2025-11-07

**Authors:** Rafał Warchulski, Krzysztof Kupczak, Vojtěch Ettler, Martin Mihaljevič, Tomasz Krzykawski, Rafał Sitko

**Affiliations:** 1 Institute of Earth Sciences, Faculty of Natural Sciences, University of Silesia, Będzińska 60, 41-200 Sosnowiec, Poland; 2 Institute of Geochemistry, Mineralogy and Mineral Resources, Faculty of Science, Charles University, Albertov 6, 128 00 Prague 2, Czech Republic; 3 Institute of Chemistry, Faculty of Science and Technology, University of Silesia, Szkolna 9, 40-006 Katowice, Poland

**Keywords:** Sludge, Metal(loid)s, Leaching, Technosol, Environmental stability

## Abstract

Untreated neutral metalliferous mine drainage sludge deposited without environmental protection for over a century was studied to assess its long-term stability. The lack of long-term stability data on such untreated sludge represents a critical knowledge gap in mine waste management. We characterized the sludge’s mineralogy, chemistry, and metal(loid) mobility using XRD, SEM–EDS, and a suite of leaching tests (deionized water, 0.01 M CaCl_2_, and pH-static extractions), supported by geochemical modeling. Key findings show that even after ~ 100 years, the sludge’s phase composition and metal(loid) content remain largely unchanged, with high concentrations of As, Cd, Pb, and Zn comparable to those in the overlying Technosol. These metal(loid)s form their own phases (e.g., cerussite, smithsonite) or are adsorbed onto goethite and clays, limiting their release under the sludge’s natural pH (6.3–7.1). Leaching is primarily confined to the sludge–Technosol interface, where the Technosol’s low pH (~ 4) promotes mobilization of Cd, Pb, and Zn (e.g., up to 342 mg kg^−1^ Zn and 278 mg kg^−1^ Pb in CaCl_2_ extracts from the Technosol). These findings are consistent with PHREEQC modeling. A remediation scenario involving raising the Technosol pH to ≥ 7 (e.g., by liming), combined with metal(loid)-binding amendments (amorphous Mn oxide, biochar, zeolite), could reduce metal(loid) migration from the sludge to the Technosol by ~ 99.9% (especially for Cd, Pb, and Zn) and significantly decrease their bioavailability to plants. This study provides the first field-based evidence that untreated neutral mine drainage sludge can remain geochemically stable for over a century, and suggests remediation strategies to ensure environmental safety of such historical mine residues.

**Supplementary Information:**

The online version contains supplementary material available at 10.1007/s10653-025-02865-3.

## Introduction

Sludge, resulting from mine drainage, is one of the primary wastes generated during mining activities since historical times. Depending on the mineralization of the ore, sludge carries various metals and metalloids (e.g., Johnson & Hallberg, 2005), which are present in the water pumped from mines due to the leaching of ores. Consequently, the diversity and concentrations of metal(loid)s in sludge depend on the mineralogical composition of the deposits, with mine water pH being a key factor. Depending on its value, acidic, alkaline, or neutral mine drainage may occur. Acid mine drainage (AMD) is mostly associated with sulfide mineralization, the oxidation of which, when exposed to air, water, and microorganisms, leads to an increase in hydrogen ion activity (Blodau, 2006; Neculita et al., 2007; Nordstrom et al., 2015; Pierre Louis et al., 2015). Alkaline mine drainage (AlMD) is characteristic of carbonate ores with low sulfur content (Banks et al., 2002). Neutral mine drainage (NMD) occurs in ores containing sulfides, but the initially reduced pH of mine water is then neutralized by leaching of carbonate minerals present in the ore (Banks et al., 2002; Motyka et al., 2017). All three types of mine drainage are known as carriers of high concentrations of metals and metalloids, e.g., Zn, Mn, As, Pb, Cd, and Cu (Beauchemin et al., 2010; McCann & Nairn, 2022; Nordstrom et al., 2000, 2015; Safira et al., 2024; Viadero et al., 2006; Wang et al., 2013).

Contemporary mine drainage water is treated using active or passive mechanisms (Rakotonimaro et al., 2017 and references therein). In the case of active methods, the pH of drained water is raised by alkaline agents, oxidants, or biological treatment (e.g., Johnson & Hallberg, 2005; Neculita et al., 2007; Skousen et al., 2000). As a result, metals and metalloids precipitate from water as hydroxides, carbonates, and sulfates (Johnson & Hallberg, 2005; USEPA, 2014; Younger, 2018). Passive approaches include anoxic limestone drains (ALD), successive alkalinity production systems/vertical flow ponds (SAPS/VFPs), biological treatment methods, e.g., wetlands, biochemical reactors (BCRs), and permeable reactive barriers (PRBs) (Johnson & Hallberg, 2005; Neculita et al., 2007; USEPA, 2014). Due to the large volumes of sludge generated by mining activities, its remediation or proper management represents a significant challenge, increasingly studied due to environmental concerns and the potential for reuse. In recent years, numerous applications for sludge have been identified, e.g., as a biostimulant, metal source, adsorbent, or pigment (Anwar et al., 2021; Bose & Tiwari, 2019; Chen et al., 2025; Zamfir et al., 2025).

The formation process and properties of modern sludge after active and passive treatment are well understood (e.g., Amanda & Moersidik, 2019; Beauchemin et al., 2010; Macías et al., 2017; Viadero et al., 2006). Few studies have examined sludge weathering and stability. Safira et al. (2024) characterized As- and Mn-rich sludge from neutral mine drainage treatment and observed negligible release of these elements in leaching tests. Similarly, Mehdaoui et al. (2025) reported that arsenic-rich NMD treatment residues aged ~ 20 years in the field remained largely stable and non-hazardous, with limited metal(loid) release under standard TCLP (Toxicity Characteristic Leaching Procedure), SPLP (Synthetic Precipitation Leaching Procedure), and FLT (Field Leaching Test) leaching protocols. These studies focused on treated sludges, shorter timescales, and only a narrow pH spectrum. This leaves a substantial knowledge gap regarding the century‑long behavior of untreated sludge and its present-day stability under a broad pH range, which is a key factor governing the mobilization of metal(loid)s from sludge (Domènech et al., 2002; McDonald et al., 2006). Such information is critical in waste management for assessing the sludge’s potential long‑term environmental impact and opportunities for its mitigation. This study aims to fill this knowledge gap using data from the abandoned Bibiela Mine in Poland. This location is an excellent site model to study the long-term weathering and soil-forming processes related to deposited sludge, as it was quickly abandoned in 1917, and the sludge ponds were exposed to these natural processes for over 100 years in the absence of protective measures or remediation efforts. The study provides a field-scale perspective by examining an untreated NMD sludge deposit over an exceptionally long natural weathering timeframe, thus offering the first evidence of its prolonged geochemical stability, surpassing previous research focused on sludge waste.

To achieve the assumed goal, we analyzed the sludge's phase and chemical composition and the mobility of metal(loid)s based on a broad spectrum of leaching tests. Overall, analyses allowed to: (i) identify metal(loid)s in the sludge and the overlying Technosol and determine the phases hosting these metal(loid)s; (ii) describe the pH-dependent leaching of metal(loid)s in Technosol and sludge; (iii) estimate the bioavailability of the metal(loid)s from the Technosol; (iv) suggest remediation scenarios.

## Location & geology

The Bibiela Mine began operations in 1889 and worked continuously until 17th June 1917. Exploitation focused on secondary iron mineralization, from the weathering of primary Mississippi Valley Type (MVT) deposits (Labus et al., 2021). The primary mineralization of these ores is composed of Zn-, Fe-, Pb-, and, less often, Cd-sulfides. Secondary mineralization consists of carbonates, sulfates, oxides, and hydroxides of the same metals (Cabała, 2009; Swęd & Duczmal-Czernikiewicz, 2019). The gangue consists mainly of carbonates accompanied by clay minerals and quartz (Cabała, 2009; Swęd & Duczmal-Czernikiewicz, 2019). The ores of the Silesian-Krakow area concentrate high levels of metal(loid)s. In addition to Fe, Pb, and Zn, they also contain Cd, Tl, As, and Mn (Table 1).

**Table 1:** Concentrations of selected metal(loid)s in MVT ores and minerals from Silesia–Krakow area

Element	Concentration	Material	References
Cadmium	up to 10.000 mg kg^−1^	Sphalerite	Mayer and Sass-Gustkiewicz (1998); Viets et al. (1996)
	700–5400 mg kg^−1^	Ores	Kucha et al. (2001)
	1283–5850 mg kg^−1^	Ores	Cabała (2009)
	> 1000 mg kg^−1^	Gangue	Cabała (2009)
Thalium	up to 500 mg kg^−1^	Sphalerite	Górecka (1996)
	100–1000 mg kg^−1^	Sphalerite + Fe sulfides	Mayer and Sass-Gustkiewicz (1998); Viets et al. (1996)
	< 1000 mg kg^−1^	Marcasite ores	Cabała (2009)
	< 90 mg kg^−1^	Sphalerite ores	Cabała (2009)
Arsenic	up to 20.000 mg kg^−1^	Marcasite ores	Cabała (2009)
	up to 10.000 mg kg^−1^	Galena + Fe sulfides	Viets et al. (1996)
Manganese	up to 10.000 mg kg^−1^	Ores enriched in clay minerals	Cabała (2009)

**Fig. 1:**
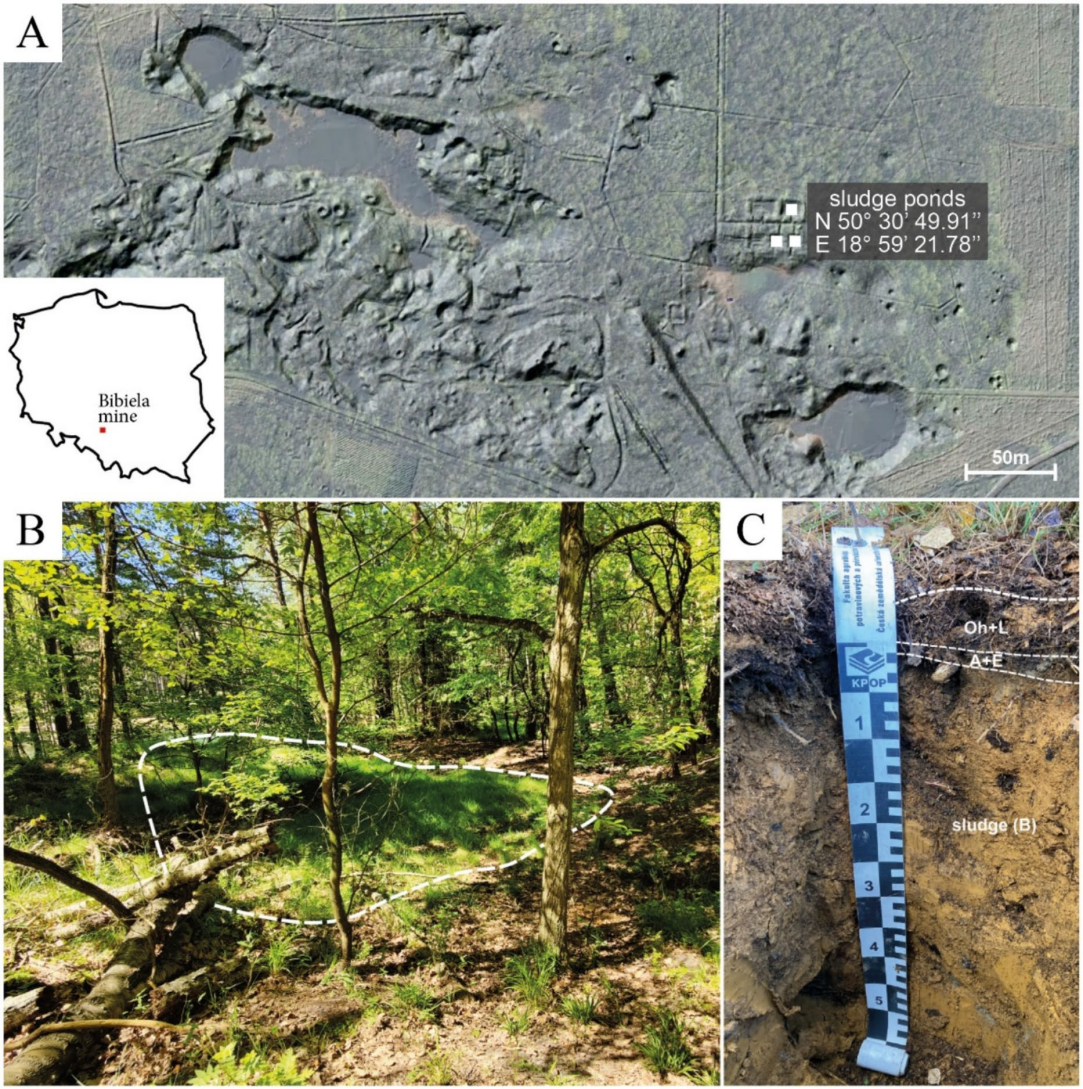
**A** satellite image with LIDAR data (geoportal.gov.pl) of the Bibiela mine area. The white squares mark the locations of the sampling profiles; **B** a photo showing the current condition of the sludge ponds (dotted line); **C** a photo of one of the excavations (BBI) within the former sludge ponds

Throughout its history, the Bibiela Mine faced constant flooding issues due to two independent aquifers (Labus et al., 2021): (i) first, related to sands and sandy clays, which occur in the overburden of the ore-bearing layer and reach the surface; (ii) the second, found in karst and fracture types associated with Triassic limestone formations below the ore layer. The existence of these aquifers necessitated the constant dewatering of the workings. The drainage system was based on a system of adits, which used electric pumps that discharged water into five interconnected sludge ponds (Fig. 1A; Labus et al., 2021). In these ponds, oxidation processes and deposition of suspended materials took place.

Considering the presence of aquifers, the co-occurrence of sulfides and carbonates in the ore, and the high concentrations of metal(loid)s in the ores, it may be assumed that neutral metalliferous mine drainage occurred in the Bibiela locality. This assumption is confirmed by data from the modern (closed in 2020) Olkusz-Pomorzany Mine and the contemporary drainage from the Bytom Mine (closed in 1989), exploiting the same ores, where the pH of the pumped water was between 6.92 and 8.26, and it was contaminated with As, Cd, Pb, Mn, and Fe when compared to guidelines from the European Drinking Water Directive (EUR-Lex, 1998; Kupich & Girczys, 2017; Motyka et al., 2017; the directive does not include Zn).

After the Bibiela Mine was flooded in 1917, the sludge ponds were abandoned and left for weathering and soil-forming processes. The area formerly occupied by the Bibiela Mine is currently governed by the Świerklaniec Forest District and is subjected to forest management.

## Methods

### Sampling & preparation

The location of the sampling points was selected based on literature data, satellite imagery, and LIDAR data (Fig. 1A), as well as field work (Fig. 1B). Three profiles were excavated to a depth of approximately 60 cm (Fig. 1C) within the individual sludge ponds (Fig. 1A). In each profile, five samples were collected from the following layers: Technosol Oh + L horizon (+ 5–0 cm; + 3–0 cm; + 2–0 cm in subsequent profiles); Technosol A + E horizon (0–2 cm); sludge (B horizon) samples from three depths (2–20 cm; 20–40 cm and > 40 cm). Approximately 60 cm was chosen as the target depth based on field observations, ensuring recovery of the entire Technosol, the surficial weathered zone of the sludge, and the underlying unaltered sludge.

For further analyses, the samples were air-dried at approximately 20 °C and homogenized by gently disaggregating and sieving to < 2 mm (mesh size according to international standard; ISO, 2006). The < 2 mm fraction of each sample was thoroughly mixed to ensure homogeneity and then split into subsamples for different treatments. One split was pulverized for preliminary bulk chemical screening using portable X-ray fluorescence (PXRF). Based on the results, samples from profile BB-I were selected for further investigation due to the high metal(loid) content. Ground samples were used for chemical analyses; unground samples were subjected to leaching tests and embedded in resin discs for scanning electron microscopy with energy-dispersive spectrometry (SEM-EDS). Samples manually ground in an agate mortar were used for powder X-ray diffraction (PXRD) analyses.

### Chemistry

All bulk chemical analyses were accompanied by quality‑assurance/quality‑control (QA/QC) procedures. For each method, samples were analyzed in duplicate or triplicate according to internal laboratory protocols to assess precision, and results were validated using certified reference materials (NIST SRM 2709a, 2710a, 2711a) along with procedural blanks to assess potential contamination. All bulk chemistry results, together with QA/QC data, are presented in Tables S1–S3.

The preliminary chemistry of all samples was determined using a portable XRF (pXRF; Thermo Scientific Niton XL3t GOLDD) with a Thermo Scientific sample holder, in AllGeo calibration mode, and an acquisition time of 4 × 30 s (total 120 s) at Charles University in Prague, Czech Republic. The major elements of the BBI profile samples were determined at the Faculty of Science and Technology, University of Silesia in Katowice, by energy‑dispersive X‑ray fluorescence (EDXRF) using an Epsilon 3 spectrometer (PANalytical, Almelo, The Netherlands). Trace elements were analyzed with ICP‑OES/ICP‑MS (Inductively Coupled Plasma Optical Emission Spectrometry/Mass Spectrometry; on an Agilent 5110 and a Thermo Scientific iCAP Q, respectively) at Charles University in Prague, Czech Republic. Before analysis, samples underwent multi‑acid digestion with HF (hydrofluoric acid), HClO_4_ (perchloric acid), and HNO_3_ (nitric acid). All the acids used in the dissolution procedure were reagent grade (Merck, Germany). The carbon and sulfur contents in the BBI profile samples were determined at the Faculty of Natural Sciences, University of Silesia in Katowice, using an ELTRA CS‑530 analyzer.

### Mineralogy

All five samples from the BBI profile were subjected to detailed petrological and mineralogical investigations. The phase composition of the samples was determined at the Faculty of Natural Sciences, University of Silesia in Katowice, by PXRD using the PANalytical X'Pert PRO PW3040/60 equipped with a Co Kα1 radiation source and an Fe filter to reduce Kβ radiation. An X'Celerator detector was used. The analyses were performed over 5–90° 2θ, at 40 kV and 40 mA. For quantitative phase composition, Rietveld refinement was performed. HighScore Plus software and the PDF‑4 + database were used. Crystal morphology and elemental distributions among phases were examined using SEM–EDS (Phenom XL) at the Faculty of Natural Sciences, University of Silesia in Katowice.

### Leaching tests

In the leaching tests QA/QC included the use of procedural blank leachates, duplicate leached samples, and certified water reference materials (NIST SRM 1643f and 1640a) to ensure precision and accuracy of the ICP‑MS measurements (Tables S4–S6). Details on the equipment used for the leaching tests are provided in the Descr. S1. Three leaching tests that differed in contact time and leaching solution were conducted at Charles University in Prague, Czech Republic. Deionized water leaching was performed to classify sludge according to EU legislation, 0.01 M CaCl_2_ leaching was used to determine the bioavailability of metal(loid)s from the Technosol and sludge, and pH-static leaching tests were used to verify metal(loid) mobility in various pH scenarios. The chemistry of leachates was analyzed with ICP-MS. The deionized water leaching test was performed in accordance with EN 12457-2 (1999). For this purpose, a 4 g sample was agitated with deionized water at a liquid/solid ratio of 10 l kg^−1^ and shaken for 24 h. The sludge sample BBI-3 was selected for this test as it contained the highest metal(loid)s concentrations. All five samples from the BBI profile layers were leached with a CaCl_2_ solution (0.01 M) at a liquid/solid ratio of 10 l kg^−1^. The samples were subjected to leaching for 2 h. The pH-static leaching test was performed on the BBI-3 sample according to EN 14997 (2015) in a liquid/solid ratio of 10 l kg^−1^. The test was performed on 11 subsamples in the pH range of 2–12 and was preceded by ANC and BNC (acid–base neutralizing capacity) analysis (Table S7). Subsamples taken from sample BBI‑3 weighing 4 g were washed with deionized water together with acid (HNO_3_) or base (NaOH) added at 0.1, 1, and 5 M depending on the target pH. The samples were monitored throughout the experiment, and their pH was adjusted by adding acid or base. In addition, a test was performed on a sample at natural pH (i.e., without an acid/base addition) and three blank samples containing: deionized water; deionized water adjusted to pH 2 with acid; and deionized water adjusted to pH 12 with base.

### Solubility calculations

The PHREEQC-3 speciation-solubility code was used to determine the speciation of elements in solution and the possible supersaturation of soil pore waters with respect to the solid phases (Parkhurst & Appello, 2013).

## Results & discussion

### Environmental stability of sludge

#### Chemistry

Preliminary chemical data for all three profiles are available in Table S1. Although some differences in metal(loid) contents between the profiles are observed, the concentrations are in the same order of magnitude. Among the metal(loid)s, four elements reach concentrations of environmental concern. Their concentrations decrease in the order Pb > Zn > As > Cd (Table S1). Based on preliminary chemical data, detailed analyses were performed on the samples from the BBI profile, which contained the highest concentrations of metal(loid)s, thus representing the “worst‑case” scenario.

Detailed chemical analyses (complete data in Tables S2 and S3) showed that the samples from the BBI profile mainly comprise SiO_2_, Al_2_O_3_, Fe_2_O_3_, and C (Table 2). All samples are chemically similar, except for the C content, in which Technosol samples (BB1-2; Table 2) are enriched. Elevated concentrations of As, Cd, Pb, and Zn were detected in all investigated samples (Table 2). Of these, the highest concentrations are reached by Pb (2–8.7 wt.%) and Zn (0.7–4 wt.%) (Table 2). In case of the BBI profile, the highest concentrations of metal(loid)s are found in the shallow sludge sample (2–20 cm; BBI-3) sludge sample: 678 mg kg^−1^ As, 208 mg kg^−1^ Cd, 8.7 wt.% Pb, and 4.02 wt.% Zn (Table 2). The chemical similarity of deeper sludge samples (20–40 cm and > 40 cm) across all profiles (Table S1; Table 2) suggests that weathering is probably limited to the shallow sludge. Metal(loid) concentrations in the Bibiela sludge after > 100 years are similar to or higher than those in contemporary sludge treated with calcium carbide residue (CCR) from Bytom (~ 20 km south of Bibiela; data for untreated sludge are unavailable), produced from the same ores. The sludge from Bytom contains 1.7–4 wt.% of Zn (cf. 1.82–4.02 wt.% in the BBI profile) and 0.05–1 wt.% of Pb (cf. 5.44–8.72 wt.% in the BBI profile) (Table 2; Kupich & Girczys, 2017) indicating a high environmental stability of metal(loid)s in the Bibiela sludge. The Technosol contains lower concentrations of metal(loid)s than the sludge. This is particularly evident for sample BBI-1. However, even in this case, the concentrations are high: 224 mg kg^−1^ As, 65 mg kg^−1^ Cd, 2 wt.% Pb, 6870 mg kg^−1^ Zn (Table 2).

**Table 2:** Bulk chemical and phase composition of the investigated Technosol and sludge from the BBI profile

		BBI-1	BBI-2	BBI-3	BBI-4	BBI-5
		Technosol	Technosol	sludge	sludge	sludge
P_2_O_5_	wt.%	0.3	0.27	0.23	0.23	0.24
SiO_2_		28	25.4	21.4	25	24.2
TiO_2_		0.68	0.67	0.59	0.83	0.9
Al_2_O_3_		9.07	11.1	9.39	14.9	15.1
Fe_2_O_3T_		25	38.7	40.3	38.2	39.9
MnO		0.23	0.23	0.55	0.32	0.23
CaO		1.75	0.41	2.5	0.36	0.37
MgO		0.2	0.11	0.61	0.15	0.17
K_2_O		0.88	0.9	0.76	1.2	1.25
Stot.		0.33	0.25	0.14	bd	0.09
Ctot.		32.1	9.56	3.06	1.59	0.97
TOC		32.1	9.54	1.65	1.34	0.76
As	mg kg^−1^	224	593	678	714	714
Cd		65	39	208	112	94
Pb		20,000	54,600	87,200	55,900	54,400
Zn		6870	14,900	40,200	18,800	18,200
Quartz	wt.%	40	30.5	28.0	18.5	14.5
Goethite		49.5	44.5	31.0	44	50
Kaolinite		4.5	20.5	10	18.5	19.5
Illite		5	3	2.5	11	10.5
Cerussite		1	1.5	9.5	5	5
Dolomite		nd	nd	15	nd	nd
Smithsonite		nd	nd	4	nd	nd
Gypsum		nd	nd	nd	nd	0.5
Sphalerite		nd	nd	nd	3	nd

bd, below detection limit; nd, not detected; _T_, all the iron has been recalculated to Fe_2_O_3_; tot., total concentration, TOC, Total Organic Carbon

#### Mineralogical composition

Based on PXRD analyses (Table 2; diffractograms are shown in Fig. S1), all samples are composed of: quartz (SiO_2_), clay minerals (kaolinite [Al_4_[Si_4_O_10_](OH)_8_)] and illite [K_0.65_Al_2.0_[Al_0.65_Si_3.35_O_10_](OH)_2_]), goethite (α— FeOOH), and cerussite (PbCO_3_). In addition, dolomite (CaCO_3_), smithsonite (ZnCO_3_), gypsum (CaSO_4_), jarosite (KFe_3_(SO_4_)_2_(OH)_6_), and sphalerite (ZnS) occur in some samples. The Technosol samples contain, relative to the sludge, more quartz (35.3 vs. 16.5 wt.%) and fewer clay minerals (16.5 vs. 23.7 wt.%) (Table 2). The goethite content varies slightly between samples (44.5–50 wt.%; Table 2). The phase composition of the shallow sludge sample (BBI-3) is intermediate between the Technosol and deeper sludge in terms of quartz and goethite content (Table 2). Sample BBI-3 is distinguished by the highest content of cerussite (9.5 wt.%) and the appearance of dolomite and smithsonite (Table 2).

**Fig. 2:**
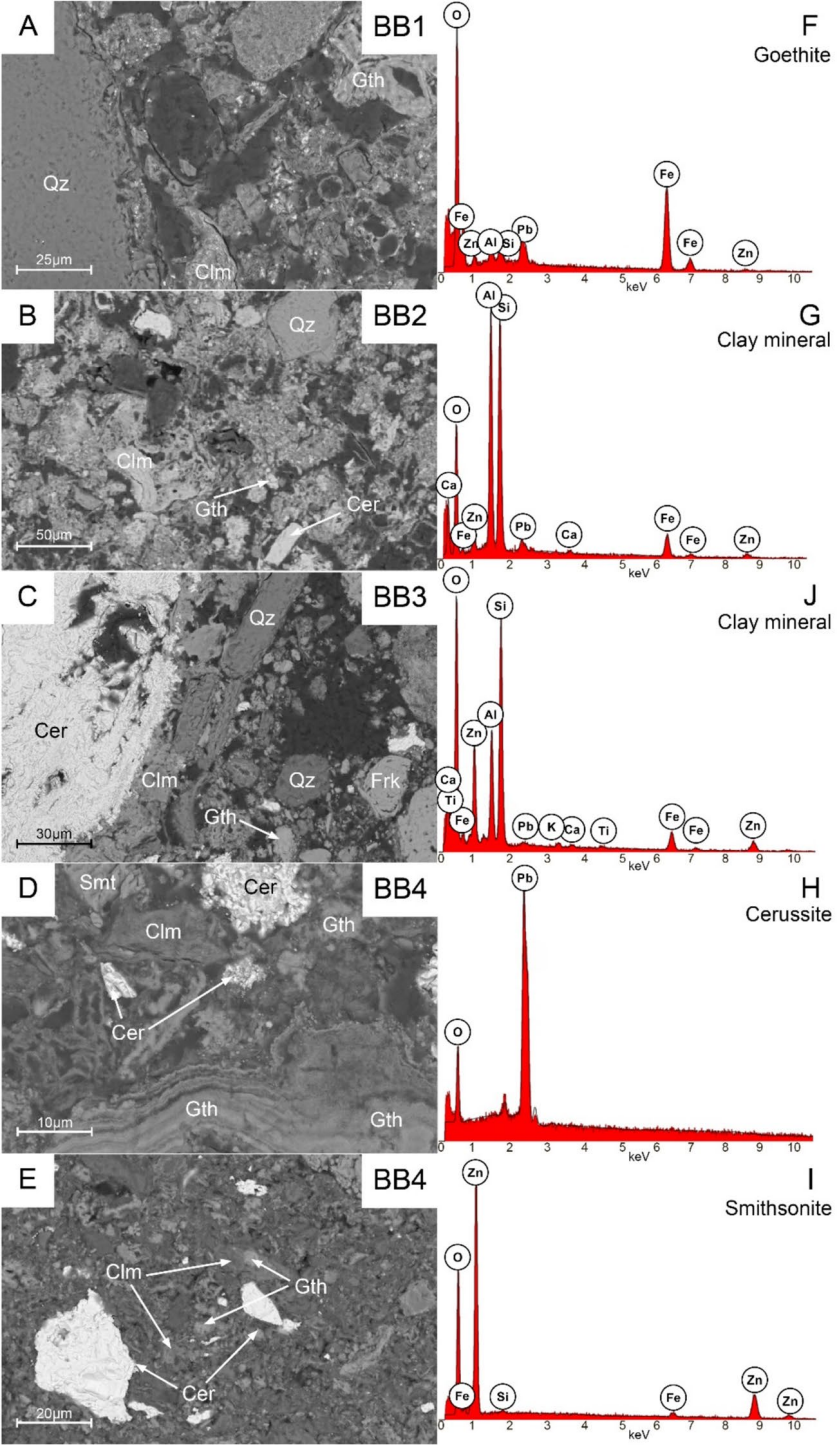
BSE (back-scattered electrons) images of Bibiela samples with EDS of selected phases. Abbreviations: Cer—cerussite, Clm—clay mineral, Gth—goethite, Frk—franklinite, Qz—quartz, Smt—smithsonite (abbreviations according to Warr, 2021)

The SEM-EDS analyses confirmed the phase composition of the analyzed samples derived from PXRD (Table 2); however, due to small grain sizes and common intergrowths, phase identification was often difficult. Based on these observations, the Technosol (Fig. 2A, B) is composed of quartz grains up to several hundred µm in size, goethite (up to 100 µm) containing structural substitutions or submicroscopic inclusions rich in Si, Zn (up to 4.53 wt.% ZnO), Pb (up to 16.52 wt.% PbO), Mg and Al (Fig. 2F), and a kaolinite‑type clay mineral (Table 2) enriched in Fe (up to 24.54 wt.% Fe_2_O_3_), Zn (up to 12.55 wt.% ZnO), and Pb (up to 5.25 wt.% PbO) (Fig. 2G, J). Additionally, isolated irregular cerussite grains up to a few tens of µm in size (Fig. 2H), baryte (BaSO_4_), dolomite, and pyrite/marcasite (FeS_2_) were observed.

In the shallowest sludge sample, quartz (up to several tens of µm), goethite (up to 20 µm), and clay minerals (aggregates up to several tens of µm; Fig. 2C) are frequent and show substitutions similar to those in the Technosol. Large cerussite crystals characterize this sample compared to the Technosol; they reach up to 300 µm in length (Fig. 2C). Additionally, Fe‑oxide of franklinite composition (ZnFe_2_O_4_), smithsonite (Fig. 2I), dolomite, and pyrite/marcasite were found.

Both deeper sludge samples comprise aggregates of mixed fine‑grained phases (most < 10 µm in diameter) with isolated quartz grains up to several tens of µm. Larger grains enabled identification of goethite, clay minerals, smithsonite, cerussite (Fig. 2D, E), and a single K‑feldspar grain.

Based on SEM–EDS and PXRD analyses, Pb is mostly bound in cerussite, kaolinite‑type clay minerals, and goethite, while Zn is hosted in kaolinite and goethite, and more rarely in smithsonite and franklinite (Fig. 2). The host phases for Cd and As could not be resolved. Cd concentrations are likely below the SEM–EDS detection limit. For As, identification is complicated by spectral overlap between As Lα lines and Mg Kα. However, given the sorption properties of kaolinite (Mohapatra et al.,  2007) and goethite (Mamindy‑Pajany et al., 2009), these phases are plausible hosts for Cd and As.

Studies on the phase composition of untreated mine‑drainage sludge resulting from AMD or NMD are scarce owing to contemporary regulations on mine‑water treatment. However, data for treated drainage confirm that the sludge composition from Bibiela was not significantly altered for more than 100 years of weathering. Goethite is a typical component of treated AMD and NMD sludge (Liu et al., 2019; Murad and Rojík, 2005; Sekula et al., 2018). Kaolinite has been reported in sludge (e.g., Amanda & Moersidik, 2019) and, together with illite, in the context of high resistance to AMD‑induced weathering (Galan et al., 1999).

The low intensity of weathering processes in the sludge, as suggested by chemical data, may reflect the low permeability of the material. From Technosol to sludge, we observe a decrease in quartz content, replaced by a higher proportion of clay minerals (Table 2), and a reduction in grain size (Fig. 2). Both the presence of clay minerals (e.g., Carcione et al., 2019) and the reduction in grain size (e.g., Díaz‑Curiel et al., 2024) can strongly reduce the permeability of the studied samples and limit infiltration by rainwater.

#### pH-static leaching

**Fig. 3:**
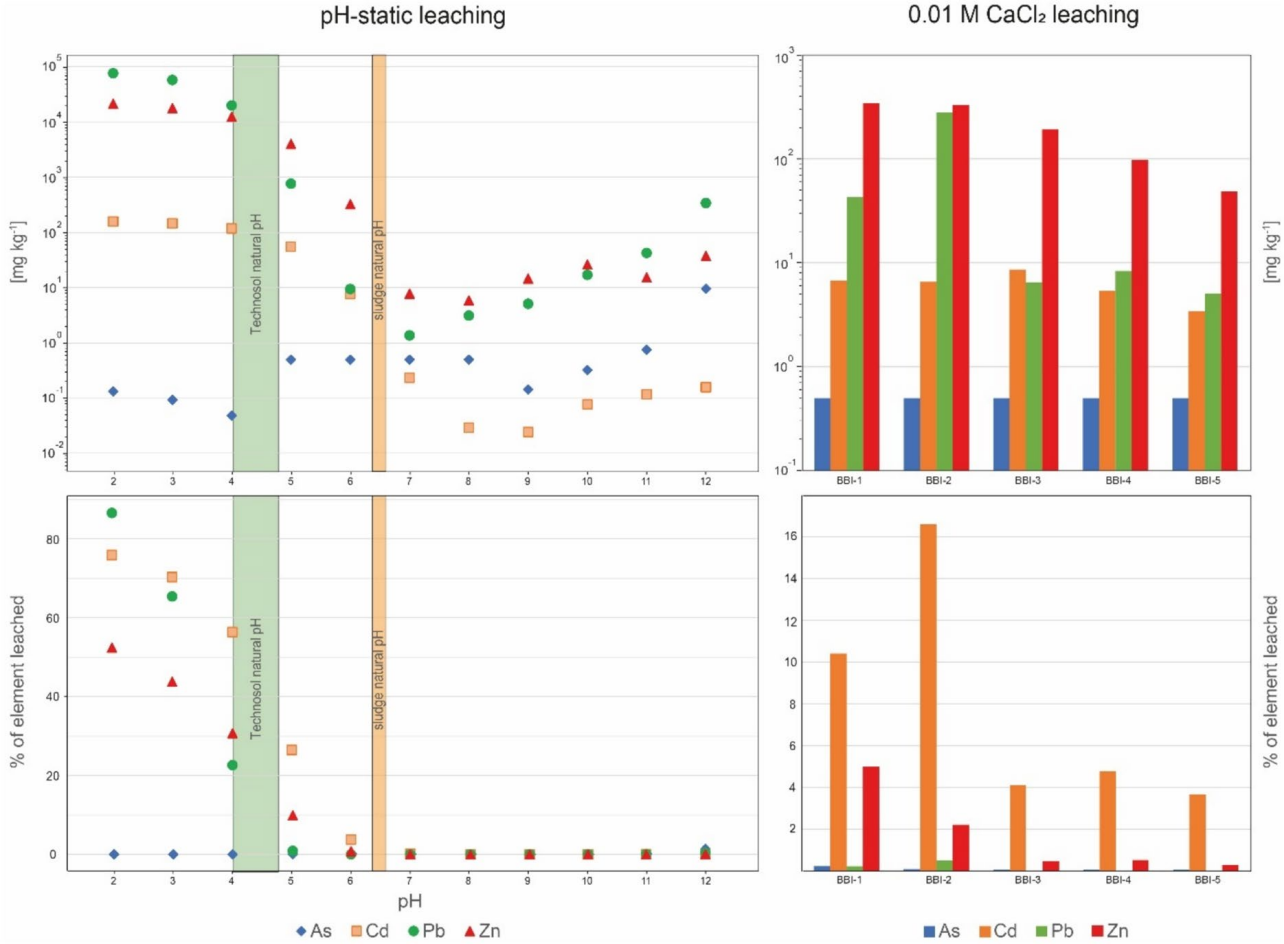
Concentrations of selected metal(loid)s in the leachates after pH-static and 0.01 M CaCl_2_ leaching tests and the percentage of selected metal(loid)s leached from the samples during these tests

The pH‑static leaching tests are used to study the emission of metal(loid)s under various environmental conditions (e.g., Cappuyns & Swennen, 2008; Ettler et al., 2015; Jarošíková et al., 2017). For the investigated sludge, as environmental pH decreases, metal(loid) leaching increases rapidly, reaching 158 mg kg^−1^ for Cd (75% of the total Cd in the sample), 7.5% for Pb (87% of the total Pb in the sample), and 2.1% for Zn (52% of the total Zn in the sample) at pH = 2 (Fig. 3). The lowest metal(loid) concentrations in the leachates occur at pH 6–11 (Fig. 3). At higher pH, concentrations in the leachates increase again, although this effect is much less pronounced than at low pH (Fig. 3). Across the pH range, Pb and Zn exhibit the highest concentrations in the leachate (Fig. 3). However, Cd is the most easily mobilized (Fig. 3), except at pH = 2, where Pb shows the highest mobilization.

The pH range with the weakest leaching of metal(loid)s from the sludge encompasses its natural pH (7.10 with deionized water; 6.26 with 0.01 M CaCl_2_; Fig. 3). Although metal(loid) concentrations in the leachates are highest at pH = 2 (a value unlikely under natural conditions, except under human gastric conditions following ingestion; Stamatopoulos et al., 2021), they remain significant at pH 3.98–4.73 (e.g., 1.97% Pb, 1.12% Zn, and 117 mg kg^−1^ Cd at pH = 4.10). This pH interval reflects the natural pH of the Technosol (Fig. 3) and thus conditions at the Technosol–sludge interface, indicating the zone where sludge weathering is currently most efficient.

#### Solubility controls

The complete results of the PHREEQC calculations of saturation indices for possible solubility‑controlling phases are available in Table S8. Based on these results, in the pH range 2–4 the leachates are undersaturated with respect to any As‑, Cd‑, Pb‑, and Zn‑bearing phases (Table 3). However, SEM–EDS analyses indicate that metal(loid)s may occur as structural substitutions and/or submicroscopic inclusions in other phases, i.e., goethite and clay minerals (kaolinite, illite). Goethite is predicted to be stable across the entire pH range investigated (2–12) (Table 3), and thus can act as a metal(loid) sink even at low pH. Supersaturation with respect to kaolinite is reached only at pH ≥ 6. Cerussite, identified in the analyzed samples (Table 2; Fig. 2H), is predicted to be stable over pH 5–11. As‑ and Cd‑bearing phases were not identified by PXRD or SEM–EDS. PHREEQC calculations indicate that these minerals are stable only at high pH (> 9; Table S8); however, their presence was not confirmed in residues from the pH‑static leaching tests.

**Table 3:** Saturation indices of selected solubility‑controlling phases as calculated by PHREEQC for leachates from the pH‑static leaching test on sample BBI‑3

	pH	1.96	3.12	4.07	5.02	5.98	6.92	7.09 (N)	7.91	8.91	9.91	10.9	12.0
Phase	Composition												
Goethite	FeOOH	***0.69***	***1.32***	***2.01***	***0.58***	***2.71***	***5.22***	***4.30***	***6.35***	***7.26***	***7.03***	***5.90***	***4.46***
Kaolinite	Al_2_Si_2_O_5_(OH)_4_	− 7.97	− 4.26	− 11.1	− 4.76	***3.28***	***5.53***	***3.82***	***5.51***	***6.91***	***5.10***	− 0.09	− 5.16
Illite	K_0.6_Mg_0.25_Al_1.8_Al_0.5_Si_3.5_O_10_(OH)_2_	− 13.9	− 8.93	− 16.3	− 8.45	***1.32***	***4.43***	***2.82***	***5.30***	***8.68***	***7.56***	***0.96***	− 5.83
Cerussite	PbCO_3_	nd	nd	nd	***0.10***	− 0.03	***0.50***	***0.72***	***1.12***	***1.32***	***1.56***	***1.12***	− 0.31
Hydrocerussite	Pb_3_(CO_3_)_2_(OH)_2_	nd	nd	nd	− 2.14	− 2.07	− 0.14	***0.91***	***2.39***	***3.90***	***5.56***	***5.46***	***3.26***
Zincite	ZnO	− 9.44	− 7.17	− 5.34	− 3.77	− 2.77	− 2.41	− 1.43	− 0.62	***1.17***	***1.69***	***1.38***	***1.13***
Hydrozincite	Zn_5_(OH)_6_(CO_3_)_2_	nd	nd	nd	− 11.4	− 7.31	− 6.22	− 2.03	***1.44***	***8.48***	***9.22***	***5.26***	− 0.15
Willemite	Zn_2_SiO_4_	− 12.9	− 8.62	− 5.23	− 2.48	− 0.84	− 0.29	***1.73***	***3.24***	***7.41***	***8.35***	***6.45***	***4.38***
Franklinite	ZnFe_2_O_4_	− 7.49	− 3.98	− 0.77	− 2.05	***3.20***	***8.58***	***7.73***	***12.6***	***16.2***	***16.3***	***13.7***	***10.6***
Otavite	CdCO_3_	nd	nd	nd	− 2.0	− 1.3	− 1.1	− 0.5	− 0.7	***0.2***	***1.1***	***0.5***	− 0.6

Supersaturation (SI > 0) of the leachates with respect to the corresponding solid phases is indicated in italics and bold. Abbreviations: nd—no data; mineral formulas are reported as in PHREEQC

The PHREEQC results suggest pH‑dependent changes in metal(loid) mobilization during the pH‑static leaching of sludge. Supersaturation with respect to kaolinite and cerussite—the main hosts of Pb and Zn (Fig. 2)—coincides with the pH interval showing the lowest metal(loid) concentrations in leachates. At pH > 7, the leachates become supersaturated with respect to numerous Pb‑, Zn‑, and Cd‑bearing phases (Table 3; Table S8). These phases can therefore act as solid sinks at the sludge’s natural pH, lowering metal(loid) mobilization. At the Technosol’s natural pH, only goethite is predicted to act as a metal(loid) sink, which helps explain the comparatively higher metal(loid) concentrations in leachates at this pH and their elevated bulk contents in Technosol samples (Table 2), despite the low mobilization from sludge at its natural pH.

#### CaCl_2_ leaching

Leaching tests with 0.01 M CaCl_2_ are used to estimate the actual mobilization of elements and their plant bioavailability in highly contaminated soils and sediments (e.g., Ettler et al., 2007; Novozamsky et al., 1993; Sahuquillo et al., 2003). They are also commonly used to determine soil pH (e.g., Houba et al., 2000); for the investigated samples, the natural pH (0.01 M CaCl_2_) ranged from 3.98 to 4.73 for the Technosol and from 6.26 to 6.55 for the sludge (Fig. 3).

Across all samples, Zn shows the highest concentrations in the leachates, followed by Pb (Fig. 3). Normalized to total content, Cd is the most readily mobilized during leaching—especially in the Technosol (mean 13.5% of total Cd leached) compared with the sludge (mean 4.21%) (Fig. 3). The results obtained for the sludge are low and similar to those obtained at natural pH during pH-static leaching, except for Cd, which reaches up to 5.34 mg kg^−1^ in the leachate (0.93 mg kg^−1^ at natural pH) (Fig. 3). The 0.01 M CaCl_2_ extraction mobilizes metal(loid)s more effectively from the Technosol: Zn up to 342 mg kg^−1^ (BBI‑1), Pb up to 278 mg kg^−1^ (BBI‑2), and Cd up to 6.73 mg kg^−1^ (BBI‑1) (Fig. 3). Accordingly, under conditions more representative of natural soils, metal(loid) phytoavailability is low in the sludge but high in the Technosol (Ettler et al., 2007; Houba et al., 2000), in contrast to the much lower mobilization observed in deionized‑water extractions. The greater release of metal(loid)s from the Technosol likely reflects its lower pH and elevated organic‑carbon content, which together enhance metal(loid) mobility. Acidic conditions (pH ≈ 4) are known to increase metal solubility and bioavailability, whereas near‑neutral conditions promote immobilization (e.g., Wang et al., 2025; Fig. 3). In addition, enrichment in organic matter (Table 2) can further promote mobilization via dissolved organic ligands: low‑molecular‑weight organic acids and other dissolved organic matter form soluble metal–organic complexes, maintaining metals in solution and increasing their phytoavailability (Li et al., 2011). In contrast, the sludge’s higher pH and predominantly inorganic matrix favor retention of metal(loid)s in less mobile forms (e.g., carbonate precipitates, adsorbed complexes), consistent with its lower CaCl_2_‑leachable fraction.

### Waste classification

**Fig. 4:**
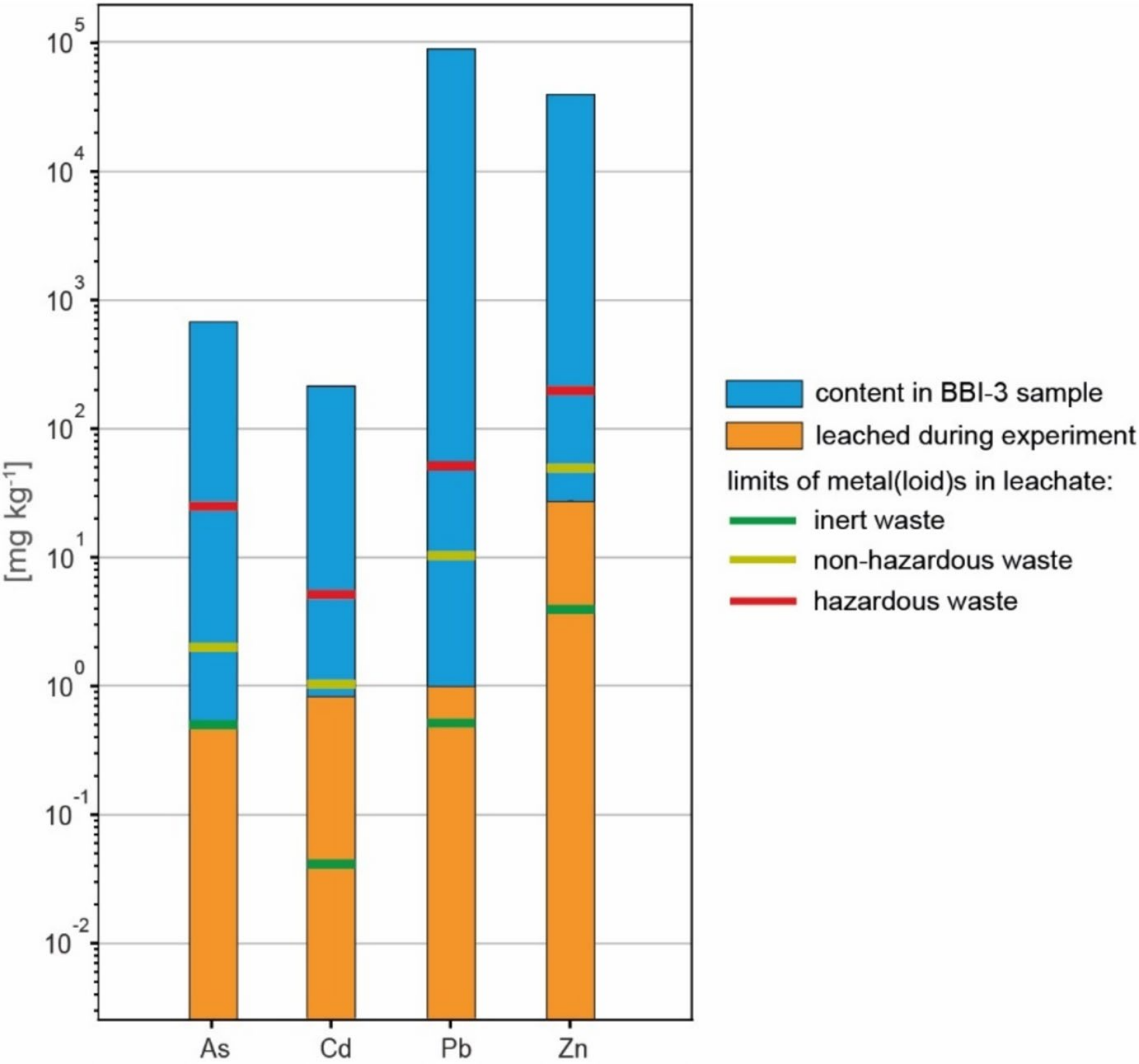
Concentrations of selected metal(loid)s in the leachate after the test according to EN-12457-2 (1999). The limit values for inert, non-hazardous, and hazardous wastes are based on the European Council Decision 2003/33/EC (EUR-Lex, 2003)

Deionized water is rarely used in soil and sediment studies because it poorly reflects actual leaching reactions in these materials; however, it is commonly used for comparison (e.g., Jarošíková et al., 2018). Leaching tests conducted in accordance with EN 12457-2 (1999) showed only slight mobilization of metal(loid)s from the sludge (Fig. 4). Zn reached the highest concentration (27.6 mg kg^−1^), whereas Cd exhibited the highest relative mobility (0.38% of total Cd leached) (Fig. 4). According to European Council Decision 2003/33/EC (EUR‑Lex, 2003), the studied sludge is classified as non‑hazardous: Cd, Pb, and Zn exceed the limit values for inert waste but do not exceed the permissible values for non‑hazardous waste. The arsenic concentration is below the limit for inert waste (Fig. 4).

According to Polish regulations on soil‑contamination assessment (Dziennik Ustaw, 2016), both Technosol samples exceed the permissible contents for the least‑restrictive soil class (IV—industrial areas, wastelands, and transport/communication areas) in terms of As (limit 100 mg kg^−1^), Cd (15 mg kg^−1^), Pb (600 mg kg^−1^), and Zn (2,000 mg kg^−1^). At the EU level, there is no harmonized regulation specifying metal(loid) contents in soils. The existing directive concerning soils receiving sewage sludge (EUR‑Lex, 1986) is more restrictive in this respect than the Polish regulations (for class IV), allowing the following contents of metals and metalloids in soil: 0 mg kg^−1^ As; 1–3 mg kg^−1^ Cd; 50–300 mg kg^−1^ Pb; 150–300 mg kg^−1^ Zn.

The high metal(loid) contents in the sludge and Technosol preclude their use for crops and (re)forestation (Dziennik Ustaw, 2016) or for reuse (Rakotonimaro et al., 2017, and references therein). At the same time, the data indicate that the sludge, in its current form, exerts limited environmental impact even over very long timescales (more than 100 years), owing to its high environmental stability.

### Remediation scenarios

The remediation strategy should recognize that metal(loid)s are mobilized from the sludge primarily at the Technosol–sludge interface due to the Technosol’s lower natural pH. The results indicate that increasing the Technosol pH to 6.9 would reduce Cd, Pb, and Zn mobilization from the sludge by 99.8–99.99% (Fig. 3). Mobilization of As may increase under this scenario; however, given its low concentration in the sludge, it is unlikely to pose a significant environmental problem. Soil pH is commonly raised using lime (CaCO_3_; Simón et al., 2010) because of its high availability and low cost.

Another challenge is stabilizing metal(loid)s within the Technosol itself. Promising results for stabilizing As, Pb, and possibly Cd in soils have been demonstrated using amorphous Mn oxide (AMO; Ettler et al., 2015; Michálková et al., 2014). Application of AMO also helps raise pH; however, given the low pH of the Technosol, direct AMO addition would lead to partial leaching because AMO is unstable at pH < 5 (Ettler et al., 2015). Moreover, the combined use of lime and AMO requires additional field‑scale testing, as AMO efficacy may decrease in some cases (Yang et al., 2021). The use of AMO does not significantly affect Zn stabilization in soils (Ettler et al., 2015). Nevertheless, given Zn’s essential role in plants (Hussain et al., 2022), its negative impact is generally lower than that of As, Cd, and Pb.

Beyond lime and AMO, other amendments show promise for immobilizing metals in soils and wastes. Biochar—a carbon‑rich product of biomass pyrolysis—can raise soil pH, supply organic functional groups for metal binding, and improve soil structure (Fathianpour et al., 2018; Sachdeva et al., 2023; Viana et al., 2025; Zhang et al., 2025). A recent meta‑analysis (Viana et al., 2025) reported that biochar alone reduced extractable Cd, Cu, Pb, and Zn in soils by ~ 24%, 33%, 31%, and 10%, respectively. Zeolites, aluminosilicate minerals with high cation‑exchange capacity, are another option (Sun et al., 2022); the same meta‑analysis (Viana et al., 2025) found that natural zeolite amendments reduced available Cd, Cu, Pb, and Zn by ~ 32%, 19%, 20%, and 39%, respectively, with zeolites particularly effective for Zn immobilization (~ 40% reduction on average). Combined use of biochar and zeolite can yield synergistic effects: co‑amendment decreased Cd availability by ~ 54% (Viana et al., 2025), and further additives (inorganic oxides/organic matter) can enhance this combination (up to 86% reduction in available Cd with mineral additives to a biochar + zeolite mix; Viana et al., 2025).

Stabilization/solidification (S/S) with cement, fly ash, or other binders is another approach that effectively locks contaminants in a hardened matrix. For example, Zha et al. (2019) showed that adding cement and fly ash to contaminated soil formed a solid matrix that greatly reduced the leachability of Pb, Zn, and Cr. The strength of S/S lies in the long‑term stability of encapsulated metals, though it can be costly and increase volume. Bioremediation approaches using metal‑tolerant bacteria have also shown success for specific elements: Ghorbanzadeh et al. (2022) reported that inoculation with two enzyme‑producing strains reduced exchangeable Zn and improved plant growth, while Zhang et al. (2022) reduced Fe, Cu, Zn, Pb, and As in leachates from Cu tailings using sulfate‑reducing and iron‑reducing bacteria. The limitation of purely biological methods is the need to maintain suitable environmental conditions (pH, nutrients, redox), and their efficacy may diminish over time if metals are not permanently sequestered (Srivastava et al., 2014).

Overall, a combined strategy—lime to adjust pH, AMO to provide high‑affinity binding sites, plus organic amendments (biochar) and selected minerals such as zeolite for specific metals—could offer a robust, multi‑mechanism approach to stabilizing the Bibiela sludge and Technosol. Each amendment has strengths and constraints: lime is inexpensive but not selective; biochar adds carbon and sorption capacity but can be less effective for oxyanion contaminants such as As; zeolites target cationic metals effectively but not As and require adequate moisture for cation exchange; AMO is excellent for As and Pb but unstable at low pH; and microbial approaches can address a broad range of metals but demand careful operational control.

### Limitations & future research directions

The present study has several limitations that must be acknowledged. The leaching experiments were conducted as batch, static tests under controlled laboratory conditions and over short timescales. While such tests offer valuable insights into potential worst‑case release scenarios, they inherently accelerate and simplify natural leaching processes and may not fully replicate the dynamic conditions encountered in the field (e.g., intermittent wetting‑and‑drying cycles, continuous percolation, or the influence of microbial activity). The use of pH‑static and single‑step extraction procedures provides a standardized framework for assessing contaminant mobility, yet long‑term leaching behavior under natural precipitation and soil processes may diverge. For instance, in‑situ diffusion limitations could further restrict contaminant release, whereas the presence of low‑molecular‑weight organic acids from rhizosphere activity might enhance the mobilization of certain metal(loid)s over time (e.g., Mizerna & Król, 2023; Ondrasek et al., 2025; Onireti et al., 2017). Moreover, the interpretations rely on the assumption of near‑equilibrium conditions, which may not be fully realized in the field. Due to the absence of long‑term historical monitoring data, we employed spatial comparisons between locations—including those involving treated mine sludge—as a proxy for temporal change. While this approach is necessary, it remains an indirect method. Analytical constraints also posed limitations: the detection limits of the applied methods hindered the direct identification of particular trace metal(loid)‑bearing phases, particularly for arsenic and cadmium. As such, our interpretations regarding their speciation—whether adsorbed onto mineral surfaces or present as minor discrete phases—remain partly inferential and should be viewed as hypotheses to be tested. Finally, while our remediation recommendations (e.g., pH adjustment and sorbent application) are grounded in experimental evidence, they have not been validated through field‑scale pilot studies. Site‑specific factors such as amendment heterogeneity, soil structure, and ecological side effects could influence the actual performance of lime, amorphous Mn oxide (AMO), biochar, or other additives. Collectively, these limitations underscore the need for field validation. Pilot‑scale trials of the lime + AMO and other amendment strategies at the Bibiela site (or similar Technosols) are needed to assess performance across seasons. Such trials would allow evaluation of pH stability over time and post‑treatment metal partitioning within the Technosol. Monitoring plant uptake and leachate chemistry before and after treatment would verify the predicted ~ 99% reduction in mobility and identify practical issues (optimal dosage, amendment longevity, unintended phytotoxicity).

## Conclusion

**Fig. 5:**
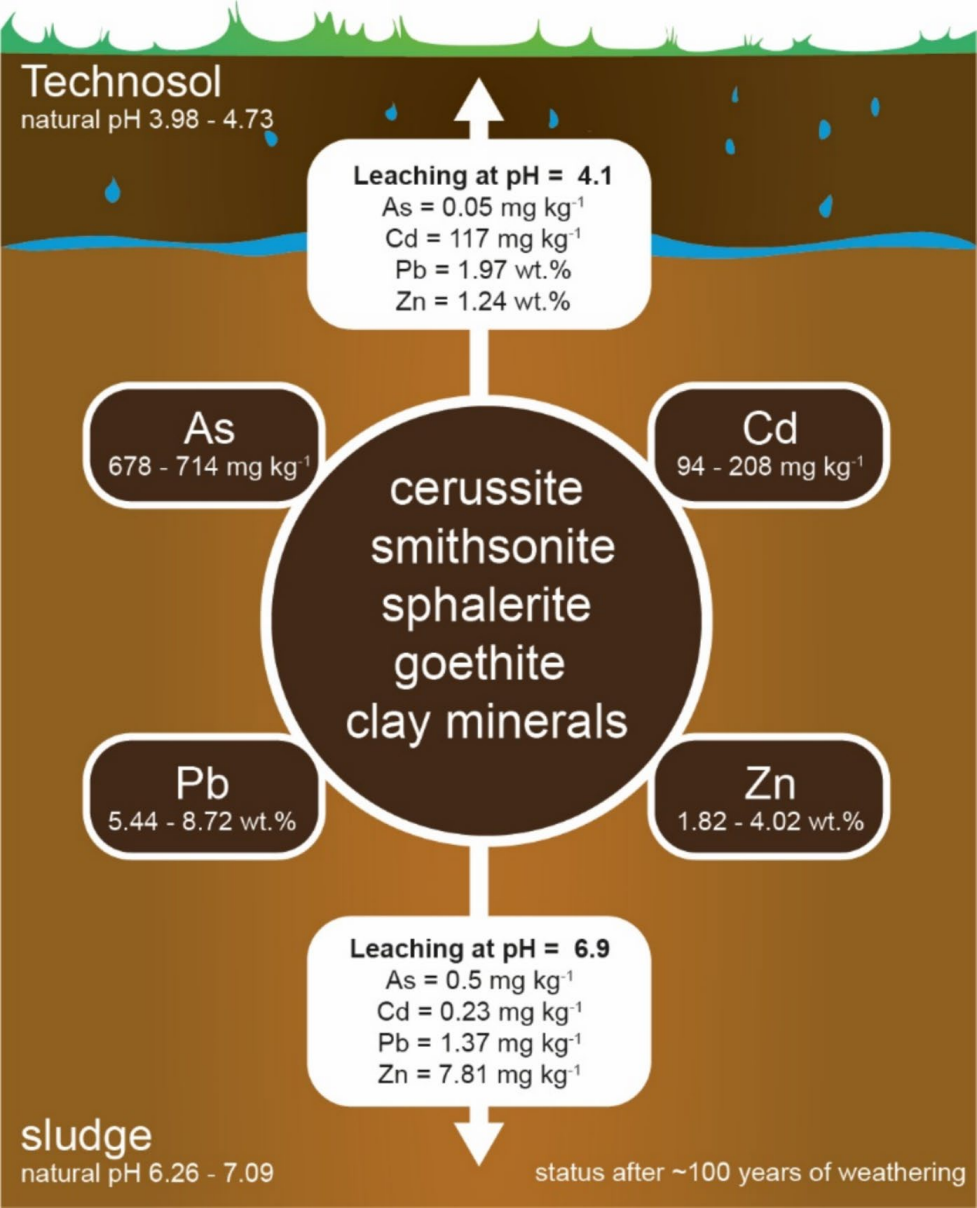
Conceptual diagram illustrating the sludge-Technosol system and metal(loid) mobility

- *Key findings* The century‑old, neutral metalliferous mine‑drainage sludge remains remarkably stable, showing no significant mineralogical or chemical alteration despite a lack of protective measures. Metal(loid) contaminants (primarily As, Cd, Pb, and Zn) persist at high levels in the sludge, but their mobility is limited by the sludge’s fine‑grained, clay‑rich matrix, which impedes water infiltration and keeps conditions near‑neutral pH (≈ 6–7). Metal(loid) release occurs predominantly at the sludge–Technosol interface (Fig. 5), where the overlying acidic Technosol (pH ≈ 4) induces localized leaching; these elements accumulate at the contact zone rather than dispersing widely.
- *Remediation efficacy* A targeted in‑situ remediation approach is recommended—raising the Technosol pH to near‑neutral (≈ 7) and adding metal(loid)-binding amendments (amorphous Mn oxide, biochar, zeolite)—to immobilize contaminants at the sludge–soil interface. Leaching tests and geochemical modeling indicate that this pH‑adjustment strategy could reduce metal(loid) transfer from the sludge into the Technosol by up to ~ 99.99%, with the binding properties of the amendments further decreasing the phytoavailability of As, Cd, and Pb in the overlying Technosol.
- *Limitations* The study was confined to a single historical site and did not include field‑scale trials, which warrants cautious interpretation of the remediation projections. Site‑specific factors (e.g., uneven amendment distribution, soil structure, seasonal pH fluctuations) could influence the success of the proposed lime + AMO treatment. Pilot field trials are needed to validate that near‑neutralization can be sustained over time and to confirm the predicted ~ 99% reduction in metal(loid) mobility, while identifying practical challenges (optimal amendment dosage, longevity, potential phytotoxicity).

This work provides the first field‑based evidence that untreated, neutral mine‑drainage sludge can remain geochemically stable over a century and underscores that historical sludge residues may be managed in situ through minimal, well‑targeted interventions (e.g., neutralizing acidic interfaces) to ensure they pose minimal environmental risk.

## Supplementary Information

Below is the link to the electronic supplementary material.

Supplementary file1 (XLSX 1769 KB)

## Data Availability

Datasets generated are available in the Supplementary Information file and are accessible in Zenodo data repository 10.5281/zenodo.16758260.
